# Successful Perioperative Management of a Patient with the Left Ventricular Assist Device for Brain Tumor Resection: Case Report and Review of the Literature

**DOI:** 10.1155/2015/839854

**Published:** 2015-03-15

**Authors:** Rashmi Vandse, Thomas J. Papadimos

**Affiliations:** Department of Anesthesiology, Wexner Medical Center, Ohio State University, Columbus, OH 43210, USA

## Abstract

Heart failure is the leading cause of death in the United States. Our increasingly aged population will contribute to an increased incidence and prevalence of heart failure, thereby augmenting the need for mechanical circulatory devices. Here we present the first successful resection of a brain tumor in a left ventricular device- (LVAD-) dependent patient with increased intracranial pressure and address pertinent perioperative anesthetic considerations and management.

## 1. Background

Heart failure continues to be the leading cause of death in United States. It is estimated to affect >5 million Americans and 550,000 new cases are diagnosed annually [1]. Although cardiac transplantation carries an excellent result for the treatment of end-stage heart failure, this option is severely limited by the number of available donor hearts. Ventricular assist devices (LAD; left (L) and (R) right) were initially developed to temporarily support the failing heart as a bridge to transplantation. Following the landmark Randomized Evaluation of Mechanical Assistance in the Treatment of Congestive Heart Failure (REMATCH) trial, which proved LVAD to be superior to any known medical therapy, LVAD is more frequently being used now as a destination therapy in patients with advanced heart failure ineligible for transplantation [2–5].

As the number of patients with long term LVAD therapy is increasing, the anesthesiologists are faced with the task of providing care to these patients for various noncardiac surgical procedures. Anesthetic considerations and perioperative management of patients with LVAD undergoing various types of noncardiac surgery have been discussed in the literature [6–12]. With this case report, we address the key anesthetic implications and issues in an LVAD supported patient undergoing elective craniotomy for resection of a brain tumor associated with increased intracranial pressure (ICP).

## 2. Case Presentation

Patient was a 60-year-old female who had an implantation of a Heart Mate II LVAD for ischemic cardiomyopathy about 2 years ago. She presented with a history of persistent severe headaches associated with confusion and balance problems. Brain imaging demonstrated four different ring-enhancing lesions within the brain. The largest one was located within the right temporal lobe measuring 4.4 × 5.3 cm. There was significant mass effect and edema in the right cerebral hemisphere including uncal herniation and 8 mm of right-to-left midline shift. Her past medical history was significant for chronic obstructive pulmonary disease (COPD), myocardial infarction, arrhythmia, congestive heart failure, and hypertension. She also had several episodes of GI bleeding in the past and hence she was maintained on a lower international normalized ratio (INR) goal of 1.3–1.8. Past surgical history included insertion of LVAD, hemiarthroplasty of R hip. Pertinent medications included furosemide, potassium chloride, albuterol-ipratropium inhaler, carvedilol, warfarin, omeprazole, fluticasone-salmeterol, trazodone, aspirin 81 mg, and sildenafil. Physical examination revealed a cachectic female who was 155 cm in height and weighed only 42.3 kg. Her Glasgow Coma Scale (GCS) was 14 and was confused at times. Her vital signs on admission were as follows: heart rate of 83 beats/minute, respiratory rate of 18–20 times/minute, blood pressure of 102/69 mm Hg, and O_2_ saturation of 93% on room air. Her neurologic examination was otherwise intact. The neurosurgery service was consulted and recommended surgical resection of the temporal brain lesion. The perioperative planning was multidisciplinary involving neurosurgery, cardiothoracic surgery, cardiology, and the anesthesiology. Her LVAD was interrogated and settings were set at a speed of 8200 rpm, pump power of 4.7, and pulse index of 6.7. She was started on dexamethasone. Her aspirin and coumadin were withheld. On the day of the surgery, her lab values were hemoglobin of 8.8 g/dL, platelet count of 9.7 × 10^9^/L which came up to 134,000 × 10^9^/L after transfusion of 2 units of platelets, INR 1.6, PTT 27, and PT 19.4. She was also given 2 fresh frozen plasma (FFP) to further decrease the INR intraoperatively. The patient was transported to the operating room by the anesthesiology team and a dedicated VAD nurse.

Intraoperative monitoring included an electrocardiogram, pulse oximetry (SpO_2_), invasive arterial pressure, and central venous pressure. Transesophageal echocardiography (TEE) was readily available. Her radial artery was cannulated prior to induction. The anesthesia was induced with 100 mcg of fentanyl, 40 mg of lidocaine, and 100 mg of propofol which was titrated slowly, followed by 50 mg of rocuronium to facilitate intubation with a size 7.0 endotracheal tube. The anesthesia was maintained with 0.8–1 MAC of sevoflurane in 50% FiO_2_ and 0.08–0.1 mcg/kg/min of remifentanil. Right internal jugular central line was placed after the induction.

In order to reduce the ICP, she was gradually placed in the reverse Trendelenburg position which she tolerated well. Furosemide 10 mg was administered and she was hyperventilated to maintain PaCO_2_ in low 30 s as confirmed by the blood gas analysis. Her mean arterial pressure (MAP) was maintained between 80 and 90 mm Hg most of the intraoperative period with only few occasional boluses of phenylephrine. She received 500 mL of crystalloids and 2 packs of FFP. She made 1700 mL of urine. Total duration of anesthesia was about 3 hours. At the end of the case, the patient's neuromuscular blockade was reversed with intravenous neostigmine (2 mg) and glycopyrrolate (0.4 mg) and was extubated deep with the return of spontaneous respiratory activity in order to avoid any coughing and sympathetic stimulation associated with the extubation. She was transported to cardiac intensive care unit in stable condition. She was slightly drowsy but was responding to commands and had a slight left sided weakness. Her GCS was 14. Her postoperative computed tomogram (CT) demonstrated small new intraparenchymal hemorrhages at the resection site. Her neurological exam, however, remained stable and she was kept under close observation with frequent neurological checks. Her repeat CT head was improving. Hence, she was started on heparin drip about 36 hours after the surgery. The patient tolerated the procedure very well and was discharged from the hospital on postoperative day 10 in stable condition.

## 3. Discussion 

This is the first case report describing successful resection of a brain tumor in an LVAD patient.

The most common neurosurgical procedure performed in LVAD patients is emergency evacuation of the intracranial hemorrhage and the outcome is usually poor. It is estimated that ICH occurs in 2.5% to 10% of patients on VAD therapy [13, 14]. Specific anesthetic considerations secondary to patient's cardiac and neurological status will be discussed in the next section.

## 4. Preoperative Management

In patients scheduled for elective surgery, thorough preoperative evaluation and optimization should be done which should address any coexisting end organ dysfunction, medications, anticoagulation status, and right ventricular dysfunction. Ideally, these patients should undergo their noncardiac surgery at centers where they received their LVAD under the supervision of the entire LVAD team (cardiac surgeon, LVAD nurses, and perfusionists, among other medical professionals) [7, 8]. The majority of these patients will be on chronic anticoagulants to minimize the risk of thrombosis. In the past, higher levels of anticoagulation were used with a target INR of 2 to 3 along with antiplatelet medications [7]. The Heart Mate II LVAD is associated with an extremely low thromboembolic risk, thereby requiring less stringent anticoagulation [6, 15, 16]. In addition to this, some patients will have acquired Von Willebrand disease secondary to the LVAD placement increasing their risk for bleeding [17]. If the surgery is elective, the patient can be bridged from warfarin to intravenous heparin preoperatively. In emergent situations, FFP can be used to reverse the effect of warfarin; however one should not aim for complete reversal of anticoagulation [6, 18]. Previous case studies have confirmed the rarity of LVAD failure despite correction of anticoagulation [6, 9, 14]. In our patient because of the intracranial surgery we were more aggressive in reversing the anticoagulation. Coumadin and aspirin were stopped preoperatively and since the surgery was relatively urgent due to brain edema and herniation, anticoagulation was reversed with FFP to decrease the INR and was also given 2 units of platelets as recommended by the neurosurgery team.

Attention must be given to electrical power needs of the device including battery back-up during transport. The care must be taken while placing the grounding pad of the electrosurgical unit so that the path of the electrical current from the unit does not go through the LVAD [7, 10]. Many of these patients also have automated internal cardioverter-defibrillators, which should be deactivated during the surgery to avoid any interference with the electrocautery unit, and external defibrillator pads should be applied [7, 10, 11]. Strict aseptic technique is required for all invasive procedures and antibiotic prophylaxis must be administered perioperatively [7, 19].

## 5. Intraoperative Management

### 5.1. Monitoring [6, 8, 12]

VAD control consoles continuously display the device output (usually an average of every four beats) which includes 4 different parameters which are as follows: pump flow in liters per minute, pump speed in RPMs, power consumption in watts, and pulsatility index (PI). As such, it provides an important parameter for the assessment and optimization of patient's hemodynamics and end organ perfusion. The Heart Mate II displays a flow based upon pump power consumption and pump rotational speed. The LVAD flow can be used as a substitute of cardiac output. However, pump flow values should be used mostly for trending any changes rather than an absolute estimate of cardiac output as there can be 15% to 20% difference between flow estimate on the display and the actual flow. Pump speed should balance adequate emptying of the left ventricle with adequate end-diastolic volume for aortic valve opening. Pump power refers to the power needed to run the motor. Typical power is 6.8 W and it is within a range of up to 25.5 W. Normally, the power will increase with speed or flow. Power that increases without an increase in speed or flow should raise suspicion for thrombus development on the rotor. The PI is a measure of the size of the flow pulse generated by the pump during the cardiac cycle. During clinical use, the PI usually ranges between 3 and 4. The PI depends on the interaction among left ventricular preload, contractility, and level of assistance from the device. A high PI indicates an increased preload, an increased ventricular contribution, or a low level of device assistance. A low PI indicates a low preload, a low ventricular contribution, a high device assistance, and inflow or outflow obstruction. In patients with first generation or pulsatile LVADs, noninvasive blood pressure measurement and pulse oximetry can be used [8, 11]. However, due to the lack of adequate pulsatile flow, hemodynamic monitoring is significantly more challenging in patients with continuous-flow LVADs. Hence, invasive blood pressure monitoring may be needed [8, 9]; pulse oximeter might not work very well as well, and serial arterial blood gas measurements or cerebral oximetry can be used as alternatives. The pulse rate on the pulse oximeter and intra-arterial blood pressure monitor reflects VAD ejection and may not be the same as the EKG derived heart rate [6, 9, 19]. Intracranial surgery is not usually associated with significant fluid shifts; hence central venous pressure monitoring is not mandatory. However depending on the patient's right ventricular function, either a central venous catheter or a pulmonary artery catheter may be used to monitor preload, RV function and for drug and volume infusion. The central line was placed because the patient had some underlying RV dysfunction and also to administer any needed vasoactive and/or inotropic agents in. TEE is recommended for procedures in which major hemodynamic changes are anticipated. For all the other cases, TEE should be immediately available [6, 10, 19].

### 5.2. Hemodynamic Goals

Intraoperative hemodynamic goals should include maintaining sufficient preload, avoiding any abrupt changes in the afterload (SVR), and maintaining RV contractility and the rate and rhythm [7]. The two most important factors that can contribute to decreased pump output are hypovolemia and increased afterload which must be avoided. LVADs are “preload dependent” and the cardiac output and stroke volume generated are limited by the volume received from the right heart [6, 9]. Even though normal or slightly increased intravascular volume is preferred [6], caution must be exercised as this can interfere with the goals of intracranial surgery [14]. It is important to judiciously follow the trends in the hemodynamics and the LVAD parameters. If there is any doubt about the patient's fluid status, TEE should be used.

The reduction in the preload can happen intraoperatively secondary to surgical blood loss, increased venous capacitance due to vasodilation induced by the anesthetic agents, institution of positive-pressure ventilation, changes in positioning especially reversed Trendelenburg, and RV dysfunction. In our case even though it is a routine practice to administer mannitol for this type of surgery to achieve brain relaxation, mannitol was not used in order to avoid fluid overload and excessive diuresis later. Additionally, positive-pressure ventilation can significantly impede venous return and preload. Hence ventilator settings were adjusted to achieve slight hyperventilation without generating unnecessarily high intrathoracic pressures. Residual RV dysfunction is common in these patients and attention must be directed at obviating the risks of RV overfilling and increasing pulmonary vascular resistance (PVR) (hypoxia, hypercapnea, overdistension of the lungs, acidosis, and light anesthesia) [10]. TEE is helpful in diagnosing RV failure and in directing therapies to decrease PVR and in initiating pharmacological support of RV dysfunction. Continuous-flow LVADs are afterload sensitive and cannot generally compensate for any abrupt increases in SVR and this can result in a diminished forward flow from the LVAD. Therefore, one must achieve an adequate anesthetic depth to avoid any sympathetic stimulation and acute increases in SVR during laryngoscopy, intense surgical stimulation, and during extubation.

Anesthetic goals from the neuroanesthesia perspective involve preserving the brain from the secondary insult by taking measures to decrease ICP, avoiding hypoxemia, hypercapnia, and hypo- and hypertension, and maximize the brain elastance to decrease the effects of retractor pressure and ischemia [14, 20]. Intracerebral perfusion should be optimized along with the conservation of cerebral autoregulation and CO_2_ responsiveness. Regarding arterial pressure, it is suggested (although without strong evidence) that the MAP be kept between 70 and 80 mm Hg [10]. Maintaining slightly higher MAP is important in patients with raised ICP in order to optimize cerebral perfusion pressure.

### 5.3. Anesthetic Agents

There is no one anesthetic technique or agent that is superior to the others. Understanding the unique physiology of the devices and the pathophysiology of the underlying heart failure and intracranial process and following the hemodynamic goals that are discussed before are crucial. Anesthetic induction should be done carefully to prevent any abrupt fall in SVR and cardiac depression. It is also important to maintain adequate depth to avoid excessive sympathetic stimulation which can increase SVR and also ICP. Fall in the SVR due to anesthetic agents can contribute to hypotension and judicious vasoconstriction should be used as necessary.

## 6. Conclusion 

Thus even though anesthetizing patients with VADs can be challenging, by meticulous preparation, monitoring, and vigilance, patients with LVAD can safely undergo some of the most complex surgeries.
